# Compassion fatigue and burnout among healthcare professionals in the ICU

**DOI:** 10.1186/cc13209

**Published:** 2014-03-17

**Authors:** M Van Mol, E Kompanje, J Bakker, M Nijkamp

**Affiliations:** 1 Erasmus MC, Rotterdam, the Netherlands; 2 Open University, Heerlen, the Netherlands

## Introduction

This study comprises a systematic review of studies focusing on the prevalence of compassion fatigue (CF) and burnout (BO) among professionals in the ICU, to indicate the size of this problem. Both CF and BO have an important impact on daily quality of life of professionals and may threaten patient care. A growing body of research suggests that BO appears to be common among ICU nurses [1] and physicians [2] due to a highly stressful work environment. However, BO might be overestimated and seen as a fashionable diagnosis [3]. The overlap of BO with CF, secondary traumatic stress (STS) and vicarious traumatisation has recently been explored [4]; CF seems to apply more to ICU staff and might be a lead in future preventive strategies.

## Methods

A review of the literature between 1998 and December 2013 was conducted using eight databases, and included the keywords CF, STS, BO, ICU, nurses and physicians. The references were screened for relevancy at title/abstract using predefined inclusion and exclusion criteria. Studies were limited to original and review articles in the English language. Two independent researchers assessed the methodological soundness of each article.

## Results

All references, retrieved from electronically database search (*n *= 1,432) and manual search (*n *= 3), resulted in 100 relevant publications for full-text screening. Subsequently, articles with only an abstract available (*n *= 28), a language barrier (*n *= 27), or prevalence not shown in percentages (*n *= 23) were removed. Finally, a sample of 22 eligible articles were appraised as methodologically sound and systematically analysed for this review. The prevalence of BO in the ICU varied from 1.2 to 49% and differed in defining a severe BO. Two studies reported the prevalence of CF, 7.3% and 40%, and five studies described the prevalence of STS in a range from 21 to 44%.

## Conclusion

The emotional price of working at the ICU can become a burden in personal life, but the size of the problem in ICU staff remains unclear. Because the decreased well-being of the professionals might negatively influence the quality of care, preventive strategies should be developed in order to alleviate the distressed. Further exploration of CF might provide sufficient starting points.
